# Supplementary material for: The Three Two-Pore Channel Subtypes from Rabbit Exhibit Distinct Sensitivity to Phosphoinositides, Voltage, and Extracytosolic pH
Source: Cells. 2022 Jun 23;11(13):2006. doi: 10.3390/cells11132006 (PMC9265530; doi:10.3390/cells11132006)
Supplement: Supplementary file 1 [file cells-11-02006-s001.zip › cells-1753631-supplementary.pdf]

Supplementary figures and table for

The three two-pore channel subtypes from rabbit exhibit distinct sensitivity to phosphoinositides, voltage, and extracytosolic pH

Xinghua Feng, Jian Xiong, Weijie Cai, Jin-Bin Tian, Michael X. Zhu

Supplementary Table S1. Primers used for reverse transcription (RT) and PCR cloning of rabbit *TPCN1* and *TPCN2* cDNA

Gene name	Primer
TPCN1	RT: 5′-GCCCTGGTCTCACGCTC-3′<br>Sense: 5′-TACCGGACTCAGATCTATGGCCGTGAGTCTCGACG-3′<br>Antisense: 5′-CTTGAGCTCGAGATCTGGGGTGACGGTCTGGGCGC-3′
TPCN2	RT: 5′-CGAGTGGCGGTCAAAGGC-3′<br>Sense: 5′-TACCGGACTCAGATCTATGGCGGAGCCCCAGGC-3′<br>Antisense: 5′-CTTGAGCTCGAGATCTGGCCTGCACAGTGGCAGGTGC-3′

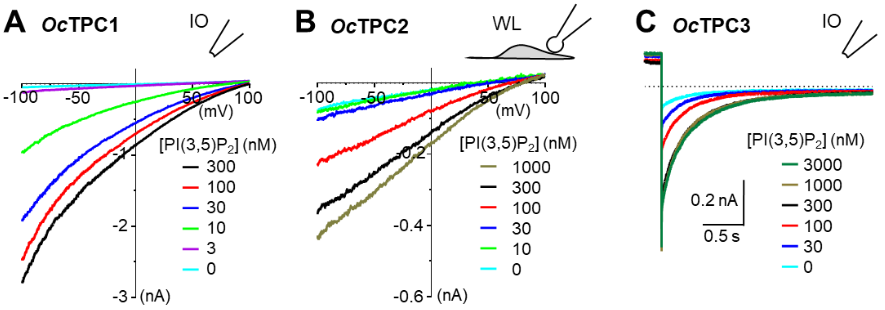

Supplementary Figure S1. Concentration dependence on PI(3,5)P_2 of rabbit TPC1, TPC2, and TPC3 - *related to Figure 2D.* HEK293 cells were transiently transfected with cDNA coding for *Oc*TPC1-EGFP (**A**), *Oc*TPC2-EGFP (**B**), and *Oc*TPC3-EGFP (**C**). (**A, B**) Representative current-voltage (I-V) curves obtained from voltage ramps for *Oc*TPC1 in inside-out (IO) and *Oc*TPC2 in whole-endolysosome (WL) patches exposed to increasing concentrations of PI(3,5)P_2 as indicated. (**C**) Representative current traces of *Oc*TPC3 in an IO patch exposed to increasing concentrations of PI(3,5)P_2 as indicated. The voltage was stepped from the holding potential at 0 mV to +100 mV for 4 s before returning to 0 mV again. Dashed line indicates zero current. For clarity, only the last 0.2 s of the +100-mV step and the tail at 0 mV are shown. Summary data are shown in Fig. 2D.

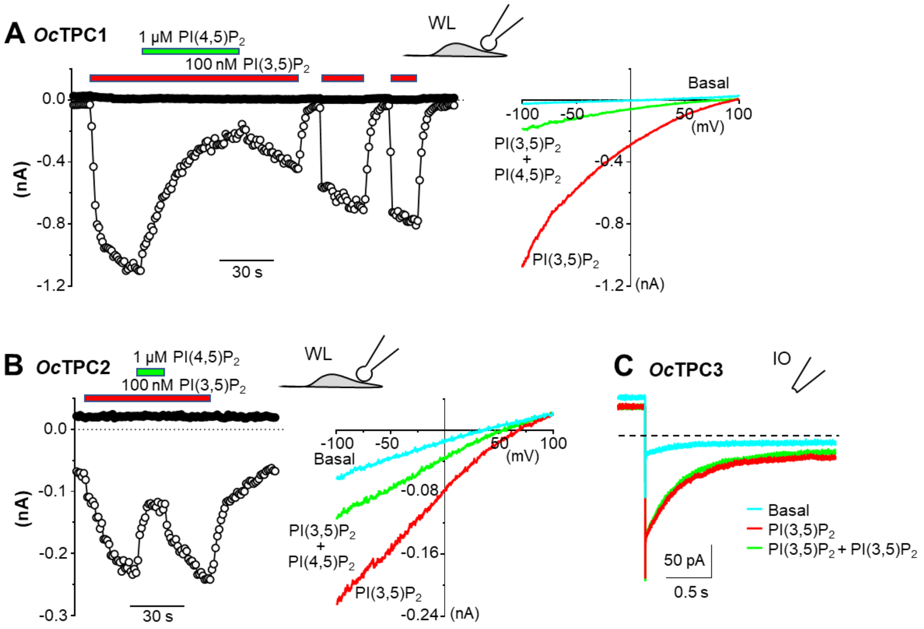

Supplementary Figure S2. PI(4,5)P_2 inhibits rabbit TPC1 and TPC2, but not TPC3 - *related to Figure 3E.* (**A, B**) Representative time courses of currents at +100 mV (*filled circles*) and -100 mV (*open circles*) recorded using voltage-ramps for whole-endolysosome (WL) patches isolated from HEK293 cells that expressed *Oc*TPC1-EGFP (**A**) and *Oc*TPC2-EGFP (**B**). The patches were exposed to 100 nM PI(3,5)P_2 and 1 µM PI(4,5)P_2 as indicated by the horizontal bars. I-V curves obtained from voltage ramps at basal (*cyan*), in the presence of PI(3,5)P_2 alone (*red*), and in the presence of both PI(3,5)P_2 and PI(4,5)P_2 (*green*) are shown to the right of the time course traces. (**C**) Representative current traces detected in an inside-out (IO) patch excised from an HEK293 cell that expressed *Oc*TPC3-EGFP, when the patch was unstimulated (basal, *cyan*), exposed to 100 nM PI(3,5)P_2 (*red*), and exposed to 100 nM PI(3,5)P_2 plus 1 µM PI(4,5)P_2 (*green*). The voltage was stepped from the holding potential at 0 mV to +100 mV for 4 s before returning to 0 mV again. Dashed line indicates zero current. For clarity, only the last 0.3 s of the +100-mV step and the tail at 0 mV are shown. Summary data are shown in Fig. 3E.
